# Treatment with Akebia Saponin D Ameliorates Aβ_1–42_-Induced Memory Impairment and Neurotoxicity in Rats

**DOI:** 10.3390/molecules21030323

**Published:** 2016-03-08

**Authors:** Yongde Chen, Xiaolin Yang, Tong Chen, Jing Ji, Li Lan, Rong Hu, Hui Ji

**Affiliations:** 1 Department of Pharmacology, China Pharmaceutical University, 24 Tongjia Xiang, Nanjing 210009, Jiangsu, China; chenyongde1123@163.com (Y.C.); cpu07415chentong@126.com (T.C.); 18061614261@163.com (J.J.); lanliapple@163.com (L.L.); 2 College of Pharmacy, Nanjing University of Chinese Medicine, Nanjing 210023, Jiangsu, China; xiaolinysn@126.com; 3 State Key Laboratory of Natural Medicines, Department of Physiology, China Pharmaceutical University, 24 Tongjia Xiang, Nanjing 210009, Jiangsu, China

**Keywords:** Alzheimer’s disease, Akebia Saponin D (ASD), Amyloid β (Aβ)_1–42_, cognitive impairment, neurotoxicity

## Abstract

Amyloid-β peptide (Aβ) is known to be directly associated with the progressive neuronal death observed in Alzheimer’s disease (AD). However, effective neuroprotective approaches against Aβ neurotoxicity are still unavailable. In the present study, we investigated the protective effects of Akebia saponin D (ASD), a typical compound isolated from the rhizome of Dipsacus asper Wall, on Aβ_1–42_-induced impairment of learning and memory formation and explored the probable underlying molecular mechanisms. We found that treatment with ASD (30, 90 or 270 mg/kg) significantly ameliorated impaired spatial learning and memory in intracerebroventricularly (ICV) Aβ_1–42_-injected rats, as evidenced by a decrease tendency in escape latency during acquisition trials and improvement in exploratory activities in the probe trial in Morris water maze (MWM). Further study showed that ASD reversed Aβ_1–42_-induced accumulation of Aβ_1–42_ and Aβ_1–40_ in the hippocampus through down-regulating the expression of BACE and Presenilin 2 accompanied with increased the expression of TACE, IDE and LRP-1. Taken together, our findings suggested that ASD exerted therapeutic effects on Aβ-induced cognitive deficits via amyloidogenic pathway.

## 1. Introduction

Alzheimer’s disease (AD) is a multifaceted neurodegenerative disorder featured by progressive memory and cognitive impairment [1,2,3]. The pathological hallmarks in AD brains are extracellular amyloid plaques composed of aggregated-amyloid (Aβ) peptide [4] and abnormally phosphorylated microtubule-associated tau protein [5]. Neurotoxicity Aβ hypothesis of AD. Aβ is derived from sequential endoproteolytic cleavage of a transmembrane Aβ precursor protein (APP) by type 1 transmembrane protein β-site APP cleaving enzyme 1 (BACE1) and γ-secretase complex yielding two major Aβ C-terminal variants, Aβ_1–40_ and Aβ_1–42_ [6,7,8]. Aggregate Aβ of low molecular weight has been proved to exhibit the greatest neurotoxicity. Alternatively, APP can be subjected to a non-amyloidogenic processing. In the non-amyloidogenic pathway, α-secretase cleavage precludes Aβ generation, releasing a soluble protein called sAPPα [9], which has been reported to have neurotrophic and neuroprotective properties [10,11]. The past decade has witnessed the discovery of multiple proteases have been shown to be capable of cleaving Aβ, including neprilysin (NEP) [12,13], insulin-degrading enzyme (IDE) [14,15] and endothelin-convertingenzyme-1 (ECE-1) [16,17]. Likely participate in regulating the steady-state levels of Aβ, IDE, a major soluble protease the degradation of extracellular Aβ in the brain, is also has the ability to remove the cytoplasmic products of APP. Many studies suggest that IDE expression responds to Aβ accumulation [18], overexpression of which attenuates brain plaque formation. Conversely, overexpression of IDE attenuates brain plaque formation, prevents premature death in a transgenic AD mouse model [19].

Many studies suggest that prolonged infusion of synthetic Aβ into the brain can cause learning and memory deficits in animals [20]. However, effective treatment or cure for AD to against Aβ neurotoxicity is still unavailable yet [21,22,23]. A complex mixture of herbs In addition, herbal medicines make a valuable contribution to the drug discovery process, the underlying mechanism of action.

Dipsacus asper wall, belonging to Dipsacaceae, is one of the most versatile Chinese herbal drugs usually growing in moist fields and mountain [24,25]. Having been used as a tonic, an analgesic and anti-inflammatory agent for hundreds of years, in the treatment of traumatic hematoma, rheumatic arthritis, bone fractures, low back pain and threaten abortion [26,27,28,29,30]. A previous study Dipsacus asper extract could ameliorate Aβ_1–42_-induced cognitive and memory impairment by regulates the level of Aβ deposition in the hippocampus [31]. Akebia Saponin D (ASD), as a typical compound isolated from the rhizome of Dipsacus asper wall, protect s PC12 cells against amyloid-β induced cytotoxicity [31] and attenuates amyloid β-induced cognitive deficits and inflammatory response in rats. Thus, we hypothesized that ASD therapeutic potential for AD.

In this study, we firstly observed the protective effects of ASD on spatial learning and memory in intracerebroventricularly (ICV) Aβ_1–42_-injected rats, and further investigated its possible underlying mechanisms by examining Aβ generation and degradation pathway.

## 2. Results

### 2.1. ASD Reversed Spatial Learning and Memory Impairment Induced by Aβ_1–42_

**Figure 1:**
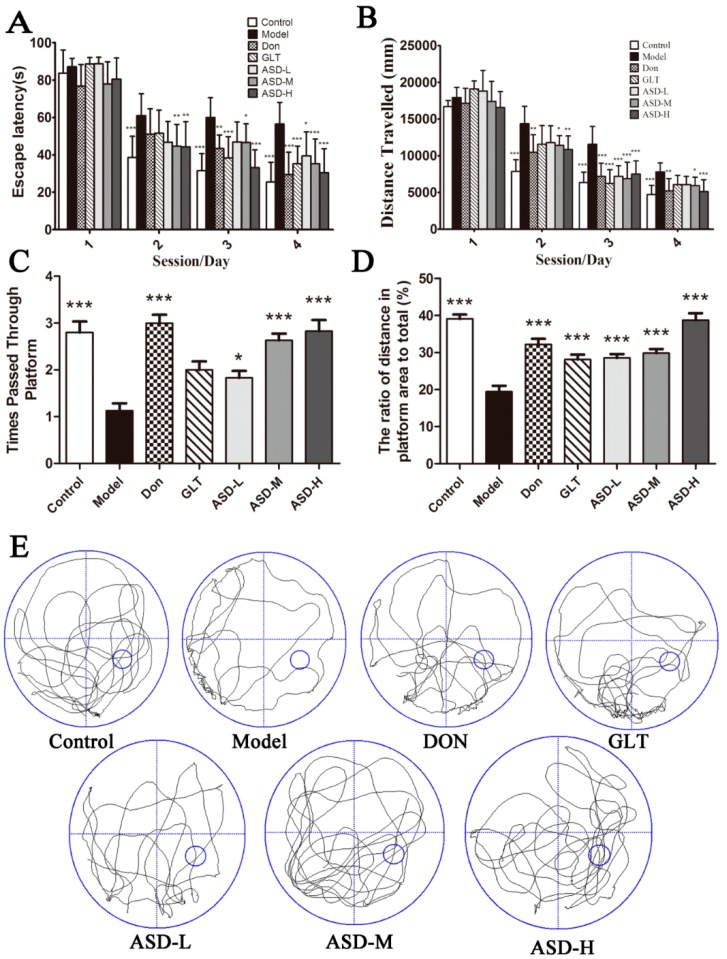
Effects of ASD on the spatial learning and memory deficits in Aβ_1–42_-induced rats evaluated by Morris water-maze test. (**A**) Changes in escape latency to reach the hidden platform during the 4-day acquisition trails and (**B**) distances traveled; (**C**) The times of former platform location crossings and (**D**) The ratio of distance in the target quadrant to total moved distance during the probe trial test are presented 24 h after the last acquisition trial; (**E**) Representative swim paths during the spatial probe test are also shown. Values shown are expressed as means ± SEM, *n* = 10, * *p* < 0.05, ** *p* < 0.01, *** *p* < 0.001 *vs.* model group.

To evaluate the effects of ASD on spatial learning and memory abilities in ICV Aβ_1–42_-injected rats, we assessed the performance of rats using Morris water maze. Regardless of different treatments, all the rats displayed progressive decrease in escape latency to reach the hidden platform. Results indicated that ICV Aβ_1–42_-injected pronouncedly increased escape latencies compared to the vehicle controls on sessions 2, 3, 4. Interestingly, rat treatment with ASD for four weeks significantly decreased escape latencies compared to the ICV Aβ_1–42_-injected group, especially at 90 mg/kg for session 3–4 and 70 mg/kg for session 4 [4 trials/rat/day for 4 days, effect of day, F (6, 69) = 264.68, *p* < 0.01; effect of group, F (6, 69) = 16.53, *p* < 0.001; effect of group-by-day interaction, F (6, 69) = 2.25, *p* < 0.001, Figure 1A]. In addition, the rats in ASD treatment groups also displayed progressive decrease in distance traveled compared to the ICV Aβ_1–42_-injected group [effect of day, F (6, 69) = 557.46, *p* < 0.001; effect of group, F (6, 69) = 17.59, *p* < 0.001; effect of group-by-day interaction, F (6, 69) = 2.92, *p* < 0.001, Figure 1B]. In the probe trial, the platform removed from the pool. The time spent in the IV quadrant and the number of platform location crossings was recorded. Rats treatment with ASD significantly increased the time s which were decreased obviously in the model group (Figure 1C), and the distances traveled were significantly increased which were decreased obviously in the model group [effect of group, F (6, 69) = 13.61, Figure 1C; effect of group, F (6, 69) = 29.68, Figure 1D]. The strategy of searching for the hidden platform was also recorded (Figure 1E). Taken together, our findings suggest that ASD may prevent significantly Aβ_1–42_-induced memory impairment in rats.

### 2.2. HE Staining

**Figure 2:**
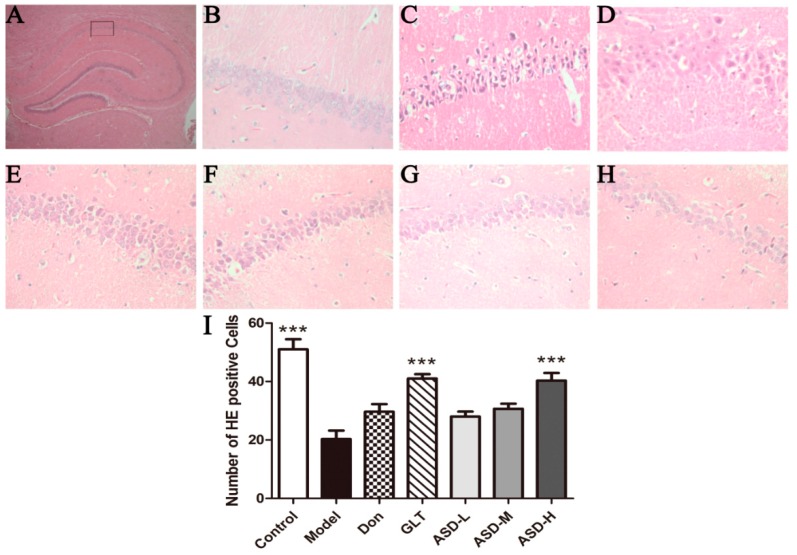
(**A**) HE staining (×400). (**B**) Control group; (**C**) Model group; (**D**) DON group; (**E**) GLT group; (**F**) ASD-L group; (**G**) ASD-M group; (**H**) ASD-H group; (**I**) Number of positive cells. Rats in control group did not show histopathological abnormalities. In Model and DON groups, cells in the hippocampal CA1 region appeared decreased in number. Furthermore, the remnants of the pyramidal cells were arranged irregularly and some exhibited shrunken and irregular shape. The cells in ASD-H group were more numerous with better cell morphology and were more numerous than those in Model and DON groups, *** *p* < 0.001 *vs*. model group.

HE staining revealed no remarkable neuronal abnormalities in the hippocampus of rats in the control group. The pyramidal cells in the CA1 region were arranged neatly and tightly, and no cell loss was found. Additionally, for the control group, cells were round and intact with nuclei stained clear, dark blue (Figure 2B). However, obvious hippocampal histopathological damage was observed in the model group. The pyramidal layered structure was disintegrated, and neuronal loss was found in the CA1 region. Neurons with pyknotic nuclei and with shrunken or irregular shape were also observed (Figure 2C). These abnormalities were attenuated by DON, GLT and ASD treatment. The cells in ASD groups had better cell morphology and were more numerous than those in the Model groups, but were overall worse than those in the control group. The average number of healthy cells was highest in the control group, lower in the treated groups, and lowest in the Model group [effect of group, F (6, 20) = 17.30, *p* < 0.001, Figure 2I].

### 2.3. ASD Blocked Aβ_1–42_-Induced Production of Aβ

**Figure 3:**
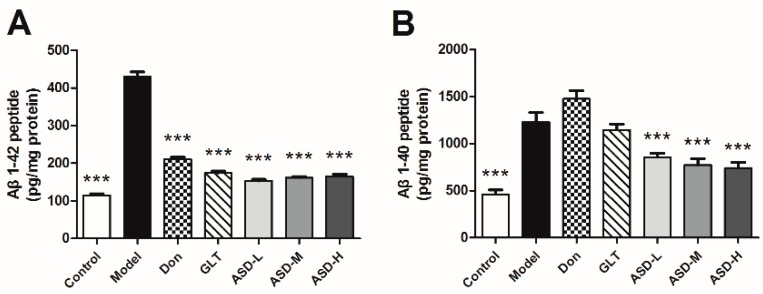
ASD blocked Aβ_1–42_-induced production of Aβ in the hippocampus of rats. (**A**) The levels of Aβ_1–40_ and (**B**) Aβ_1–42_ in this extract were quantified by ELISA assays. Values shown are expressed as means ± SEM, *n* = 10, *** *p* < 0.001 *vs.* model group.

To examine the potential mechanisms of ASD, the levels of Aβ_1–42_ and Aβ_1–40_ in hippocampus were measured by ELISA assays. As the result exhibited, there was a rise in concentration of Aβ_1–40_ and Aβ_1–42_ in ICV Aβ_1–42_-injected group compared to the corresponding vehicle control group. Of note, ASD treatment significantly decreased the level of Aβ_1–42_ and Aβ_1–40_ compared with model group [effect of group, F (6, 69) = 290.0, *p* < 0.001, Figure 3A; effect of group, F (6, 69) = 24.23, *p* < 0.001, Figure 3B]. As demonstrated by these results, ASD might regulate the generation of Aβ_1–40_ and Aβ_1–42_.

### 2.4. Effects of ASD on the Expression of Aβ Generation Proteins

**Figure 4:**
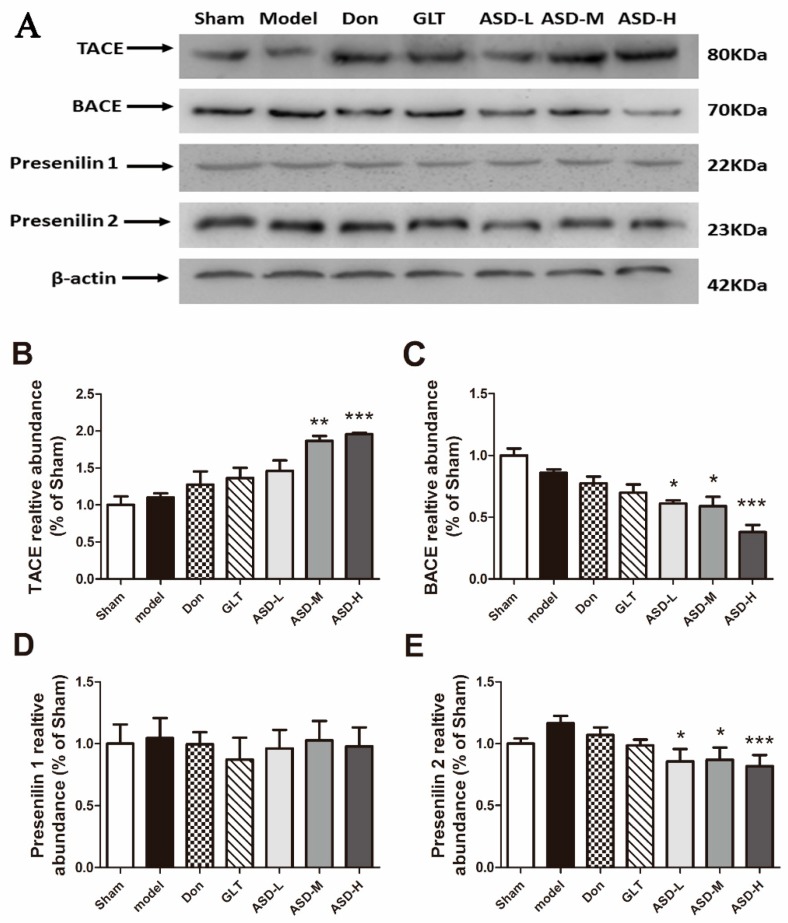
Effects of ASD treatment on the expression of Aβ generation-relation proteins (**A**) The protein levels of TACE, BACE, Presenilin 1, and Presenilin 2 were detected by western blotting. Quantification of TACE (**B**); BACE (**C**); Presenilin 1 (**D**) and Presenilin 2 (**E**) was represented as the ratio (in percentage) of the sham group. β-actin was used as a loading control. The data were expressed as mean ± SEM of three independent experiments, * *p* < 0.05, ** *p* < 0.01, *** *p* < 0.001 *vs.* hippocampus of vehicle plus Aβ_1–42_ group.

To explore the underlying mechanisms of ASD against Aβ-induced neurotoxicity by down-regulating the generation of Aβ_1–42_ and Aβ_1–40_, we examined the expression of TACE, BACE, presenilin 1 and presenilin 2 by Western blot analyses. As the results exhibited, the expression of TACE was significantly increased in the hippocampus of rats treated with ASD [effect of group, F (6, 20) = 9.960, *p* < 0.001, Figure 4B]. The expression of BACE and presenilin 2 was decreased [effect of group, F (6, 20) = 13.38, *p* < 0.001, Figure 4C; effect of group, F (6, 20) = 13.38, *p* < 0.001, Figure 4E], the expression of presenilin 1 had no significant change [effect of group, F (6, 20) = 0.1369, *p* = 0.9889, Figure 4D].

### 2.5. Effects of ASD on the Expression of Aβ Degradation-Related and Transshipment-Related Proteins

**Figure 5:**
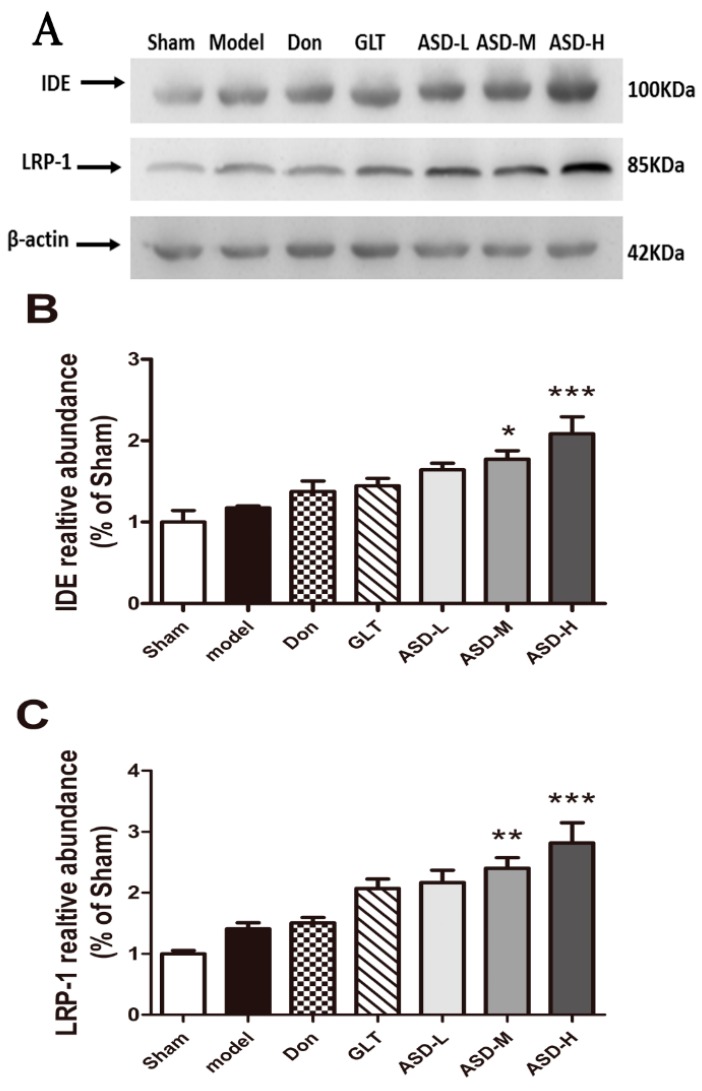
Effects of ASD treatment on the expression of IDE and LRP-1 using western blotting analysis. (**A**) The protein levels of IDE and LRP-1 were detected by western blotting. Quantification of IDE (**B**); LRP-1 (**C**) was expressed as the ratio (in percentage) of the sham group. β-actin was used as a loading control. The data were represented as mean ± SEM of three independent experiments, * *p* < 0.05, ** *p* < 0.01, *** *p* < 0.001 *vs.* hippocampus of vehicle plus Aβ_1–42_ group.

To further explore the underlying mechanisms of ASD on Aβ degradation, we examined the expression of IDE and LRP-1 by Western blot analyses. As shown in Figure 5, ASD significantly increased the expression of IDE and LRP-1 in the hippocampus of rats (*p* < 0.05 or *p* < 0.01, Figure 5A,B).

## 3. Materials and Methods

### 3.1. Animals and Housing

A total of 84 Sprague-Dawley rats of adult male, weighing between 250 and 300 g, were procured from B & K Laboratory Animal Corp. Ltd. Shanghai, China. Throughout the study period, the rats were maintained in accordance with the guidelines of National Institutes of Health Guide for Care and Use of Laboratory Animals (publication no. 85-23, revised 1985). Twelve rats were housed in each cage during the study period and were maintained under standardized conditions (22 ± 1 °C, 60% ± 10% humidity, 12 h light/dark cycle). Water was provided ad libitum.

### 3.2. Drugs and Materials

**Figure 6:**
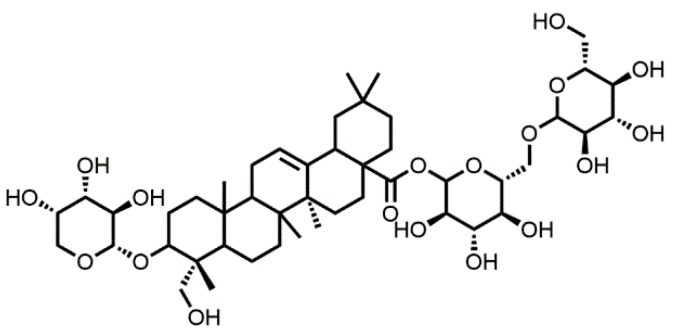
Chemical structure of ASD.

ASD (Figure 6) was prepared by Professor Zhong-Lin Yang. Isolated from Dipsacus asperoides at the Key Laboratory of Modern Chinese Medicines, China Pharmaceutical University, and and MALDI-TOF mass spectrometry revealed >93.4% purity. Commercially Aβ_1–42_ (Invitrogen, Waltham, MA, USA) was stored at −20 °C, then the peptide was dissolved in sterile normal saline at concentration of 2 μg/μL, this solution was incubated at 37 °C for 7 days before the surgery [32].

### 3.3. Stereotaxic Intracerebroventricular (ICV) Aβ_1–42_ Injection and Drug Treatment

The rats were randomly divided into seven groups: Control, Model (Aβ_1–42_), Donepezil (Aβ_1–42_ + 0.5 mg/kg donepezil) as Positive Control 1, Ginkgo leaf tablets (Aβ_1–42_ + 25mg/kg GLT) as Positive Control 2, ASD-H (Aβ_1–42_ + 270 mg/kg ASD), ASD-M (Aβ_1–42_ + 90 mg/kg ASD) and ASD-L (Aβ_1–42_ + 30 mg/kg ASD). During the surgery, rat were anesthetized with intraperitoneal injection of sodium pentobarbital (30 mg/kg) and then placed on a stereotaxic frame (SR-5, Narishige, Tokyo, Japan). The dura overlying the parietal cortex was exposed. Two burr holes were drilled through the skull in suitable locations according to the stereotaxic atlas of Watson and Paxinos (A = 3.3 mm caudal to bregma, L = 2.0 mm lateral to midline, and V = 2.5 mm below the Dural surface). In the control group, 5 μL sterile normal saline was injected bilaterally at a rate of 1 μL/min by pump, and in the other groups, 5 μL Aβ_1–42_ (2 μg/μL) was injected bilaterally. The micropipettes were left in place for 5 min to minimize back-flux of liquid [33].

The day of ICV Aβ_1–42_ injection was designated as Day 0. One day after the surgery, rodent feed and sterile distilled water were provided ad libitum. The test group, ASD (30, 90, 270 mg/kg), donepezil (0.5 mg/kg) [34,35] or Ginkgo Biloba Extract (25 mg/kg) were intragastrically administered once daily for consecutive 30 days. In control and model groups the same volume of vehicle as ASD were received.

### 3.4. Morris Water Maze Task

The Morris water maze task test was conducted to evaluate the spatial learning and memory abilities, which consisted of 4-day training and a probe trial on day 5, carried out from Day 26 to Day 30. This was performed as descried previously [36,37]. Rats were individually trained in a black circular pool (150 cm in diameter and 50 cm in height) filled to a depth of 30 cm water with temperature maintained at 25 ± 1 °C. The circular pool was divided into four imaginary quadrants (I, II, III and IV). On each training day (Day 26 to Day 29), the platform always in the middle of IV quadrant (10 cm in diameter) was submerged 1 cm below water and hidden from the rats’ view. The animals were subjected to four trials with a 1 h interval between trials. Each trial lasted for 90 s unless the animal reached the platform. If an animal failed to find the platform within 90 s, the test would be ended with the animal being gently navigated to the platform by hand for 30 s. On day 5 (Day 30), the platform was removed and the probe trial was started, during which animals had 90 s to search for the platform. The time spent in the target quadrant and the number of target crossings (*i.e.*, the quadrant where the platform was previously located) was recorded. Data of the escape latency, the time spent in the target quadrant, the number of target crossings and swimming speed were collected by the video tracking equipment and processed by a computer equipped with an analysis-management system (Viewer 2 Tracking Software, Ji Liang Instruments, Shanghai, China).

### 3.5. Hematoxylin-Eosin Staining of Neurons in the Hippocampus of Rats

On Day 28, following the last treatment, 3 rats of each group were decapitated after injection of a lethal dose of chloral hydrate. The brains were rapidly removed and immersed in 4% formaldehyde for 48 h, then removed and dehydrated in a dehydrator overnight before being embedded in paraffin. Brains were section at a thickness of 5-μm sections and then baked for 20 min. Subsequently, sections were dewaxed with xylene three times followed by washed with 100%, 95%, 90% and 85% ethyl alcohol solutions for 1 min each and washed with water. Next, sections were stained with hematoxylin, differentiated with hydrochloric acid alcohol solution, treated with 1% ammonia, and stained with eosin for 3 s. After being washed with water, and then 85%, 90%, 95% and 100% ethyl alcohol for 1 min each, sections were cleared in xylene for 2 min and cover-slipped with resin.

### 3.6. Western Blot Analysis

Rat hippocampi were washed with PBS, chopped off into small pieces, and then homogenized in ice-cold RIPA buffer (Beyotime Institute of Biotechnology Co., Ltd. Haimen, China) containing 0.5% phosphatase inhibitor, 1% phenylmethylsulfonyl fluoride. The homogenate was centrifuged at 12,000× *g* and 4 °C for 15 min the supernatant containing dissolved proteins. Equal amounts of protein were measured using Coomassie bluebased assay kit (Beyotime Institute of Biotechnology Co., Ltd.), t TACE, BACE, presenilin 1, presenilin 2, LRP-1 and IDE. Separated by 10% SDS-polyacrylamide gels, and then transferred onto a PVDF membrane. PVDF membranes were blocked 2 h at room temperature with 5% non-fat milk in Tris-buffered saline with 0.1% Tween 20(TBST). In addition, then the membranes incubated at 4 °C overnight with respective primary antibodies for anti-TACE (1:300, Santa Cruz, Dallas, TX, USA), anti-BACE (1:1000, Cell Signaling Technology, Danvers, MA, USA), anti-presenilin 1 (1:1000, Cell Signaling Technology), anti-presenilin 2 (1:1000, Cell Signaling Technology), anti-IDE (1:1000, Epitomics, Burlingame, CA, USA), anti-LRP1 (1:1000, Epitomics), or anti-β-actin (1:3000, Bioword, Nanjing, China). After washing with TBST three times, the blots were incubated with a horseradish peroxidase conjugated secondary antibody (goat anti-rabbit IgG, 1:50,000, Bioworld Technology Co., Ltd., Nanjing, China) for 2 h at room temperature. After washing with TBST five times, the antibody-reactive bands were visualized by using the enhanced chemiluminescence detection reagents (Tanon Science & Technology Co., Ltd., Shanghai, China) by a gel imaging system (ChemiScope 2850, Clinx Science Instruments Co., Ltd., Shanghai, China). The band intensity analysis was calculated by AlphaEaseFC software (Alpha Innotech, San Leandro, CA, USA).

### 3.7. Measurement of Aβ_1–40_ and Aβ_1–42_

Rat hippocampi were washed with PBS, chopped off into small pieces, and then homogenized in ice-cold PBS buffer. Containing the homogenate was centrifuged at 12,000× *g* and 4 °C for 15 min. Equal amounts of protein were measured using Coomassie bluebased assay kit (Beyotime Institute of Biotechnology Co., Ltd., Haimen, China). The levels of Aβ_1–40_ and Aβ_1–42_ were assayed using an ELISA kit (Shanghai Jianglai Bioengineering Institute, Co., Ltd., Shanghai, China) according to the manufacturer’s protocol. All reactions were performed in triplicates and recorded in 96-well microplates, using a microplate spectrophotometer.

Standard curves were prepared with synthetic Aβ_1–42_ (Bachem, Torrance, CA, USA) diluted in extracts of non-transgenic mouse forebrain prepared in parallel as described above. Each sample was analyzed in duplicate.

### 3.8. Statistical Analysis

All experimental data shown are expressed as means ± SD. All the data, unless specified, were analyzed with one-way ANOVA which were followed by Newman–Keuls tests for multiple comparisons using SPSS 11.5 software (SPSS China, Shanghai, China). Statistical significance was considered when *p* < 0.05.

## 4. Discussion

A primary pathological hallmark of AD is the accumulation of Aβ in brain [38], which is generated by sequential cleavage from the proteolytic processing of β-amyloid precursor protein (APP). APP generates various peptide species including Aβ_1–42_, a more toxic form prone to oligomerize leading to neuroinflammation [39,40,41], synaptic dysfunction [42,43], apoptosis [44,45] and memory impairment [46], all of which are observed during the progression of AD. One of the important strategies for preventing or treating AD is to interfere with the toxic Aβ species.

Here, we demonstrated that ASD prevented Aβ_1–42_-induced spatial learning and memory impairments, as evidenced by the decrease in escape latency during acquisition trials and improvement in exploratory activities in the probe trial in the MWM task, coupled with modified expression generation-related proteins (TACE, BACE, Presenilin 2), degradation-related proteins (IDE) and transshipment-related proteins (LRP-1).

In this study, rats exposed to 10μg amyloid beta (1–42) into the bilateral hippocampus presented a significant impairment in learning and memory ability. In addition, insulin degrading enzyme (IDE), an enzyme involved in Aβ clearance, can regulate the level of Aβ in hippocampus. As the results showed, treatment with ASD (90 mg/kg, 270 mg/kg) significantly increased the levels of secreted IDE (* *p* < 0.05, *** *p* < 0.001) and dose-dependently improved the learning and memory deficits induced by Aβ_1–42_. In agreement with the experiment data, we hypothesized that ASD might ultimately enhance the levels or activities of secreted IDE which led to induced clearance of Aβ in the hippocampus. Moreover, Aβ_1–40_ and Aβ_1–42_ are produced by the proteolytic processing of APP. Proteases referred to as secretase cleave at the N-and C-termini of Aβ within APP to generate Aβ. A previous study showed that the levels of Aβ_1–40_ is much higher than Aβ_1–42_ after APP cleavage, we investigated the levels of Aβ_1–40_ in hippocampus at the same time.

The results indicated that ASD can significantly reduce the levels of Aβ_1–40_ in hippocampus, along with changes in the levels of Aβ generate e related enzyme. In conclusion, we hypothesis that ASD regulate the proteolytic processing of APP, mainly regulate the α-secretase, β-secretase or Presenilin 2 to reduce the levels of Aβ_1–40_ in hippocampus.

In conclusion, we demonstrated that ASD, a saponin separated from Dipsacus asperwall, significantly improved the learning and memory abilities impaired by Aβ injury, protected neurons and markedly regulating Aβ generate-related proteins (TACE, BACE, Presenilin 2) expression. In addition, we found that ASD might promoted the Aβdegradation by regulating Aβdegradation-related proteins (IDE) expression. These findings indicate that ASD might have a therapeutic potential in AD. In the future, In the future, much work requires to be done to demonstrate the effects of ASD on the deposition of amyloid plaques, neurofibrillary tangles and other distinct neuropathological features in AD.

## 5. Conclusions

In conclusion, the present study has demonstrated for the first time that ASD exerted potently therapeutic effects on Aβ-induced cognitive deficits via amyloidogenic pathway, as evidenced by a decrease tendency in escape latency during acquisition trials and improvement in exploratory activities in the probe trial in Morris water maze (MWM). Moreover, we firstly found that ASD reversed Aβ_1–42_-induced accumulation of Aβ_1–42_ and Aβ_1–40_ through down-regulating the expression of BACE and Presenilin 2 accompanied with increased the expression of TACE, IDE and LRP-1.
